# Genetic alterations of GI‐NECs involving three main signaling pathways

**DOI:** 10.1002/cam4.5633

**Published:** 2023-01-18

**Authors:** Qiong Dai, Jinping Zhang, Weili Long, Johannes Haybaeck, Zhihui Yang

**Affiliations:** ^1^ Department of Human Anatomy Southwest Medical University Luzhou Sichuan China; ^2^ Department of Pathology The Affiliated Hospital of Southwest Medical University Luzhou Sichuan China; ^3^ Institute of Pathology, Neuropathology and Molecular Pathology, Medical University of Innsbruck Innsbruck Austria; ^4^ Diagnostic & Research Center for Molecular BioMedicine, Institute of Pathology, Medical University of Graz Graz Austria

## Abstract

**Background:**

Gastrointestinal (GI)‐neuroendocrine neoplasms (NENs) are subclassified in neuroendocrine tumors (NETs), neuroendocrine carcinomas (NECs), and mixed neuroendocrine–non‐neuroendocrine neoplasms (MiNENs). The genetic characteristics of GI‐NEN has been a hot issue in recent years, but more studies are needed to provide further details. This study aims to provide additional data about genomic characteristics of GI‐NENs and the genetic differences between NETs and NECs.

**Patients and Methods:**

Thirteen samples were selected for next‐generation sequencing (NGS) analysis with a 425‐gene panel. Microsatellite instability (MSI) and tumor mutational burden (TMB) were calculated as well as immunohistochemistry (IHC) was used to test for protein expression.

**Results:**

Genetic alterations were very common in NECs, but rare in NETs. The average TMB of NETs and NECs was 2.3 and 6.9, respectively. The TMB of NECs was significantly higher compared to NETs. The *TP53* mutation rate was significantly higher in NECs than in NETs (100% vs. 20%), other mutations involved *MTOR* (*n* = 2, 15.4%), *DDR2* (*n* = 3, 23.1%), *ERBB4* (*n* = 1, 7.7%), *BRCA1* (*n* = 1, 7.7%), *BRCA2* (*n* = 1, 7.7%), *ATM* (*n* = 1, 7.7%), and *SMAD4* (*n* = 1, 7.7%). Deep loss of *SMAD4* (1/3, 33.3%), *SDHB* (1/3, 33.3%), *RB1* (1/3, 33.3%), and *BRCA2* (1/3, 33.3%), high‐level amplification of *CRKL* (1/3, 33.3%), *CCNE1*(1/3, 33.3%), and *MCL1*(1/3, 33.3%) were found in NECs. The integrated analysis found these genetic alterations frequently involve DNA repair and cell cycle, PI3K/AKT/mTOR and TGF‐β/SMAD4 signaling pathways.

**Conclusion:**

Genetic alterations were very common in NECs and rare in NETs, and frequently involved three main signaling pathways. NEC patients harboring these genetic alterations may benefit from targeted therapy and PD‐1/PD‐L1 immunotherapy.

## Graphical abstract

Only little is known about the genetics of gastrointestinal‐neuroendocrine neoplasms (GI‐NENs), samples of 13 GI‐NEN patients were selected for next‐generation sequencing (NGS) analysis of 425 genes in this study. We found genetic alterations were frequently involved DNA repair and cell cycle, PI3K/AKT/mTOR and TGF‐ß/SMAD4 signaling pathways in NEC patients. NEC patients harboring these genetic alterations may benefit from targeted therapy and PD‐1/PD‐L1 immunotherapy.

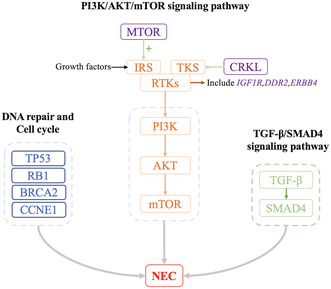

## 1 INTRODUCTION

Neuroendocrine neoplasms (NENs), which are essentially heterogeneous malignant neoplasms originating from neuroendocrine cells, occur at various sites of the body. The gastrointestinal (GI) tract is the most common site of primary NENs, including stomach, duodenum, small intestine, appendix, colon, and rectum.^1^ In recent years, due to the widespread usage of endoscopy, the incidence of GI‐NENs has increased,^2^ and the incidence of gastric NENs has even increased approximately 15‐fold.^3^ In the fifth edition (published in 2019) of the Digestive Tumors Classification of the World Health Organization (WHO), GI‐NENs are classified as neuroendocrine tumors (NETs), neuroendocrine carcinomas (NECs), and mixed neuroendocrine–non‐neuroendocrine neoplasms (MiNENs), based on morphology and proliferative activity.^3^ Proliferation rate is assessed by mitotic count and the Ki‐67 proliferation index. Values <3% of Ki67 index and <2/10 HPF (10 mm^2^) of mitotic count determines NET G1; 3–20% of Ki67 index and 2–20/10 HPF (10 mm^2^) of mitotic count, reveals NET G2; >20% of Ki67 index and > 20/10 HPF (10 mm^2^) of mitotic count, according to their morphological differentiation, is classified as NET G3 or NEC G3.^3^ Surgical resection remains the cornerstone of treatment for localized GI‐NENs, and chemotherapy is the mainstay of treatment for NEC patients in advanced stages.^4^, ^5^ A few noteworthy findings had indicated the possible genomic characteristics of GI‐NENs.^6^, ^7^ Genetic alterations may explain their clinical heterogeneity, divergence for treatment response, and prognosis.^8^ However, more studies are need to provide further details on genetic characteristics of GI‐NENs. Genetic alterations, identified through next‐generation sequencing (NGS), may provide the unprecedented opportunity to investigate genomic differences in the various histological types of GI‐NENs. Consequently, the presence of genetic changes may provide the potential for defining strategies for individualized treatment. In this study, we report the genomic results of 13 GI‐NEN cases examined by NGS, in conjunction with detailed histological subtyping, immunohistochemical, and clinicopathologic characterization. We aimed to investigate the detailed genomic characterization of GI‐NENs to explore its biologic difference between NETs and NECs.

## 2 MATERIALS AND METHODS

### 2.1 Patients and samples

In this study, 13 patients were recruited from 182 GI‐NEN cases by selection between January 2010 and December 2019. They were hospitalized in the Affiliated Hospital of Southwest Medical University. Two senior pathologists (Yang, Z.H. and Long, W.L.) reviewed and confirmed the diagnosis based on the fifth edition Digestive System Tumors Classification of WHO.^2^, ^3^ Clinical and pathological data of patients, including time of diagnosis, tumor size, location, metastatic status, histological subtypes, grades, depth of invasion, clinical stages, therapy, as well as information on recurrence, and date of death, were collected.

The study was approved by the ethics committee of the Affiliated Hospital of Southwest Medical University (KY20200079). The entire study was conducted in accordance with relevant guidelines. Upon signing the informed consent form, subjects were able to participate in this study.

Formalin‐fixed paraffin‐embedded (FFPE) blocks of the 13 patients with NENs were collected from the archive of the Department of Pathology for examination.

### 2.2 Immunohistochemistry (IHC)

Antibodies directed against AE1/AE3(PCK), chromogranin (CgA), synaptophysin (Syn), CD56, Ki‐67, P53, α‐thalassemia/mental retardation syndrome X‐linked (ATRX), death domain associated protein (DAXX), and insulinoma‐associated protein 1 (INSM1) were tested. Staining for these proteins were performed using the automated immunohistochemical staining platform of Roche BenchMark Ultra (Roche Diagnostics) or Dako Ominis (Agilent Technologies) together with the respective detection kits.

### 2.3 NGS and bioinformatics analysis

#### 2.3.1 NGS

Genomic DNA was extracted from FFPE tissues using QIAamp Kit (Qiagen, QIAGEN Cat: 56404), which concentration was determined by the Nanodrop 2000 (Thermo). Up to 2 μg of DNA was absorbed and placed into the crushing tube and placed on the holder for crushing. Beads were added for separation and purification to enter the library construction.^9^ Library quantification was tested by Qubit3.0 with a dsDNA HS Assay Kit (Life Technology), and analyzed by KAPA Library Quantification kit (KAPA Biosystems). The size distribution of libraries was measured by Agilent Technologies 2100 Bioanalyzer (Agilent Technologies).

Human cot‐1 DNA (Life Technologies) and xGen Universal blocking oligos (Integrated DNA Technologies) were added to block nonspecific binding. Customized xGen lockdown probes panel (Integrated DNA Technologies) were used to targeted enrich for 425 predefined genes. The NimbleGen SeqCap EZ Hybridization and Wash Kit (Roche) was used in hybridization reaction. Probe‐bind fragments were captured by Dynabeads M‐270 (Life Technologies). Library amplification was performed with Illumina p5 and p7 primers in KAPA HiFi HotStart ReadyMix (KAPA Biosystems), and purification by Agencourt AMPure XP beads. Libraries of different samples were uniquely labeled and clustered together for targeted enrichment. Sequencing was performed on the Hiseq 4000 NGS platforms (Illumina) with coverage depths of at least 1000x for FFPE.

#### 2.3.2 Bioinformatics analysis

Gene mapping was completed by comparing the raw sequencing data and the genomic data of Chinese individual human genomics HG19(GRCh37). Single nucleotide variants (SNVs) and short insertions/deletions (indels) were confirmed by VarScan2.^10^ The threshold of minimum variant allele frequency was set at 0.01, and the *p*‐value was set at 0.05. All SNVs/indels were annotated using ANNOVAR, and each SNV/indel was manually checked on the Integrative Genomics Viewer (IGV). Copy number variations (CNVs) were detected by in‐house‐developed software.

Tumor mutational burden (TMB) includes nonsynonymous and synonymous single base mutations and indel, excludes driver mutations and clear inactivation mutations of tumor suppressor genes. TMB value is calculating the number of somatic nonsynonymous mutations or all mutations per megabase.

Microsatellite instability (MSI) status of samples determination was reached by detecting the proportion of unstable positioning points in 52 microsatellite sites. If the proportion was higher than 40%, the result was regarded as Microsatellite instability‐High (MSI‐H).

### 2.4 Statistical analysis

The comparative analysis was performed by Chi‐square (*χ* ^2^) test using SPSS v22 (IBM SPSS Statistics), and *p*‐value <0.05 was defined a statistical significance.

## 3 RESULTS

### 3.1 Histopathological assessment

**FIGURE 1:**
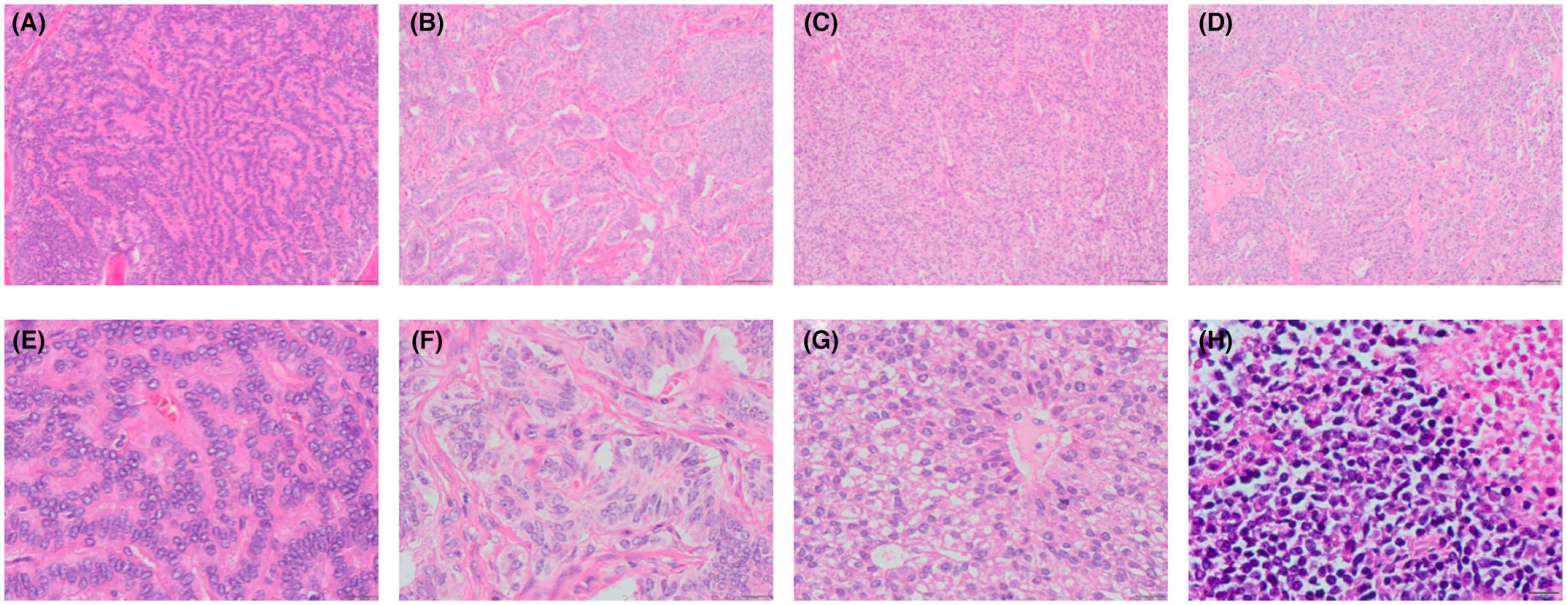
Typical morphological features of GI‐NENS. H&E staining show NETs consist of small, uniform nuclear features. They arrange in islets, cords, trabeculae, and gland formation. Cells of NECs are pleomorphic, characterized as sheets growing, mitosis and necrosis are common. A (100×) and E (400×) showed NET G1, B (100×) and F (400×) show NET G2, C (100×) and G (400×) show NET G3, D (100×)and H (400×) showed NEC.

NETs contain small, uniform nuclei with a so called “salt‐and‐pepper” chromatin. The tumor cells are usually arranged in an organoid architecture, such as islets, cords, trabeculae, ribbons, and gland formation, with minimal necrosis (Figure 1A–C, E–G). Conversely, cells of NECs are pleomorphic, with markedly atypical nuclei, sometimes with prominent nucleoli, often arranged in sheets, with extensive necrosis (Figure 1D, H).

### 3.2 IHC results

**FIGURE 2:**
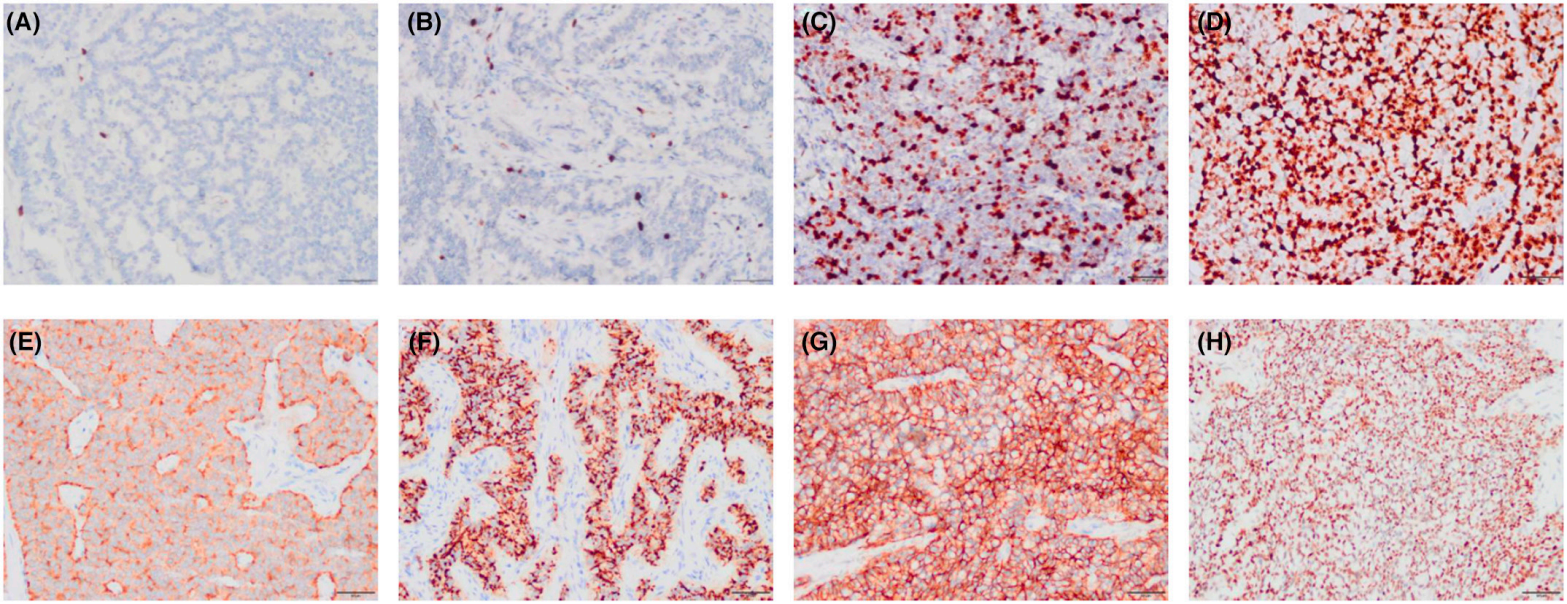
Typical immunohistochemistry features of GI‐NENs. (A–D) (200 ×) represents Ki‐67 index of NETs and NECs, which is 1%, 3%, 40%, and 80%, respectively. (E–H) (200 ×) shows positivity for Syn, CgA, CD56, and INSM1 expression in GI‐NENs.

Within all patients, the Ki‐67 index varied significantly between NETs and NECs (Figure 2A–D). The most strongly expressed IHC markers among the 13 GI‐NENs included PCK (13/13, 100%), Syn (12/13, 92.3%), CD56 (11/13, 84.6%), CgA (7/13, 53.4%), and INSM1 (11/13, 84.6%) (Figure 2E–H). No case was positive for ATRX and DAXX. The Ki‐67 index varied significantly between NETs and NECs (Figure 2). P53 positivity rate was 33.3% (1/3) in NECs.

### 3.3 Clinicopathologic characteristics of the 13 GI‐NENs cases

A total of 13 samples was detected successfully by NGS, including 10 NETs and three NECs. Thirteen patients were classified as NENs with NET G1 (*n* = 4), NET G2 (*n* = 3), NET G3 (*n* = 3), and NEC (*n* = 3). The age distribution of patients (six men and seven women) ranged from 36–62 years (average age 49.3 years). The primary sites were rectum (*n* = 7), stomach (*n* = 5), and small intestine (*n* = 1). Eight patients were treated by surgical resection and five by endoscopic forceps. The tumor size ranged from 0.5 to 5 cm (median, 1.0 cm). The depth of invasion was serosal layer (*n* = 3), muscularis (*n* = 3), and submucosa (*n* = 7). Ten cases had been evaluated for the clinical stage, three NET G3 patients could not be accurately staged (Table 1).

**TABLE 1:** Clinicopathological features of 13 GI‐NENs patients

Characteristics	Number of patients (%)
Sex	
Male	6(46.2%)
Female	7(53.8%)
Age at diagnosis	
<50 years	6(46.2%)
≥50 years	7(53.8%)
Sites	
Rectum	7(53.8%)
Stomach	5(38.5%)
Small Intestinal, appendix	1(7.7%)
Tissue types	
NET G1	4
NET G2	3
NET G3	3
NEC	3
Type of surgery	
Surgical resection	8(61.5%)
Endoscopic forceps	5(38.5%)
Size	
<2 cm	7(53.8%)
≥2 cm	6(46.2%)
Depth of invasion	
Serosal layer	3(23.1%)
Muscularis	3(23.1%)
Submucosa	7(53.8%)
Clinical stages	
I/II	9(76.9%)
III/IV	1(7.7%)
Lymph node metastasis	
Yes	2(15.4%)
No	11(84.6%)
Distant metastasis	
Yes	2(15.4%)
No	11(84.6%)
Adjuvant therapy	
Yes	5(38.5%)
No	8(61.5%)

Abbreviations: NEC, neuroendocrine carcinoma; NET G1, neuroendocrine tumor grade1; NET G2, neuroendocrine tumor grade2; NET G3, neuroendocrine tumor grade3.

### 3.4 Follow‐up results

The time of follow‐up ranged from 13 to 89 months (average 48 months). Of the 13 patients, five had recurrences and metastases in their follow‐up, three NEC patients received radiotherapy after surgery. All NEC patients relapsed and metastasized in a short time, and their average overall survival (OS) was 15.3 months. Two NET G3 patients presented with recurrence and metastasis, but the OS was longer than 5 years. Another eight patients exhibited no abnormalities during follow‐up (Table 2).

**TABLE 2:** Personal clinicopathological features of 13 GI‐NENs patients

patient	Sex	Age (years)	Site	Size(cm)	Depth of invasion	Pathological grading	Clinical stages	Lymph node metastasis	Adjuvant therapy	Overall survival (month)
1	Female	40	Stomach	5.0	Serosal layer	NET G3	^a^	Present	Chemotherapy	55
2	Male	59	Stomach	0.5	Submucosa	NET G2	I	Absent	No	49
3	Female	62	Stomach	3.0	Muscularis	NET G3	^a^	Absent	Chemoradiotherapy	89
4	Female	39	Stomach	3.2	Muscularis	NET G3	^a^	Absent	No	78
5	Male	57	Stomach	2.0	Serosal layer	NEC	II	Absent	Chemotherapy	20
6	Female	46	Rectum	0.6	Submucosa	NET G1	I	Absent	No	44
7	Male	55	Rectum	0.6	Submucosa	NET G1	I	Absent	No	84
8	Female	47	Rectum	0.5	Submucosa	NET G1	I	Absent	No	48
9	Female	53	Rectum	0.7	Submucosa	NET G1	I	Absent	No	41
10	Male	54	Rectum	0.8	Submucosa	NET G2	I	Absent	No	27
11	Male	51	Rectum	1.0	Submucosa	NET G2	I	Absent	No	57
12	Male	36	Rectum	4.0	Muscularis	NEC	III	Present	Chemotherapy	13
13	Female	42	Small Intestinal	4.7	Serosal layer	NEC	II	Absent	Chemoradiotherapy	13

^a^ No staging standard or consensu*s*.

### 3.5 Mutation differences between NETs and NECs

**FIGURE 3:**
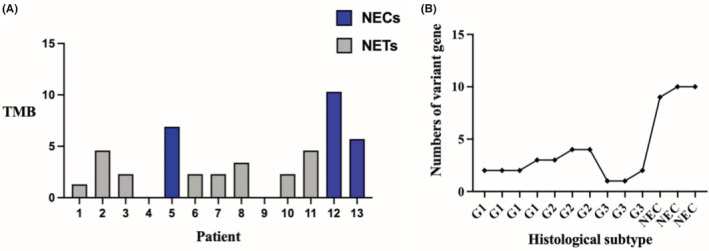
(A) The number of patients and corresponding TMB value, the TMB of all NEC patients are more than five. (B) shows the histological subtypes and their corresponding numbers of variant genes, the numbers of variation genes of NECs are significantly higher than NETs.

**FIGURE 4:**
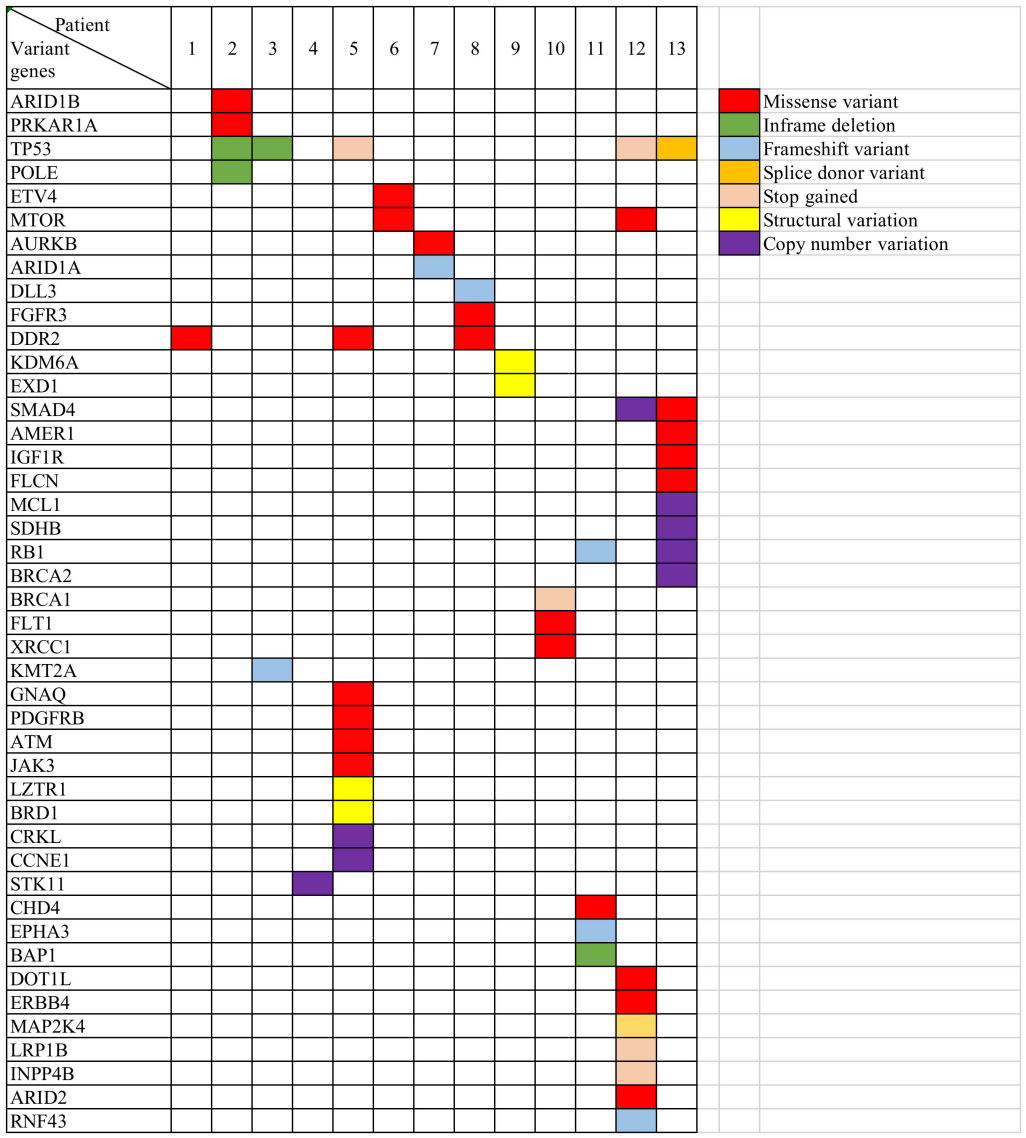
The genetic alterations of 13 GI‐NEN patients: involved genes in details.

Targeted NGS of 13 samples identified 40 non‐synonymous somatic mutations (24 missense, 6 frameshift, 4 stop‐gained, 4 deletion, 2 splice donor). Genetic alterations are known to commonly occur in NECs, but rare in NETs. The most common genomic variation in these 13 samples was *TP53* (*n* = 5, 38.4%) gene mutation. The *TP53* mutation rate was 100% (3/3) in NECs, and 20% (2/10) in NETs. About 33.3% (2/6) of *TP53* mutations was found in patients with NET G2 and G3, but it was not found in any of the patients of NET G1 (0/4, 0%). The difference of *TP53* mutation between NETs and NECs was significant (*p* < 0.05). The types of *TP53* mutations include inframe‐deletion, stop‐gained, and splice‐donor‐variant. An *RB1* mutation has been found in one NET G2 patient and one NEC patient. Other mutations of genes, such as *SMAD4* (1/3, 33.3%), *MTOR* (*n* = 2, 15.4%), *DDR2* (*n* = 3, 23.1%), *ERBB4* (*n* = 1, 7.7%), *BRCA1* (*n* = 1, 7.7%), *BRCA2* (*n* = 1, 7.7%), and *ATM* (*n* = 1, 7.7%) were found. The average TMB of GI‐NET and G3‐NEC was 2.3 and 6.9, respectively. There was no difference concerning TMB among NET G1, G2, and G3, and the value of TMB in all NET patients was less than five. However, the TMB of NECs was more than five. There were significant differences regarding TMB between NETs and NECs (*p* = 0.0009). The numbers of individual genetic alterations and TMB values of all patients are shown in Figure 3. The detailed sequencing analysis results are shown in Table 3 and Figure 4.

**TABLE 3:** The gene variations landscape of the 13 GI‐NENs patients

Patient	Pathological grading	Variation type	Variation genes	Variation site	AA change	Copy number variation	TMB
1	NET G3	Mutant	DDR2	c.371G > A	p.R124Q		1.2
2	NET G2	Mutant	ARID1B	c.2158 T > C	p.F720L		4.6
			PRKAR1A	c.1018G > T	p.V340F		
			TP53	c.652_657del	p.V218_P219del		
			POLE	c.3351_3359del	p.S1118_Q1120del		
3	NET G3	Mutant	TP53	c.706_726del	p.Y236_C242del		2.3
			KMT2A	c.9226_9263del	p.E3076Lfs*21		
4	NET G3	CNV	STK11			0.47	0.0
5	NEC	Mutant	TP53	c.586C > T	p.R196*		6.9
			GNAQ	c.1036G > A	p.D346N		
			PDGFRB	c.263C > T	p.T88I		
			ATM	c.2578G > T	p.D860Y		
			DDR2	c.1373G > A	p.R458H		
			JAK3	c.2777 T > A	p.L926H		
		SV	LZTR1	IGR (downstream BRD1) ~ LZTR1:exon15			
			BRD1				
		CNV	CRKL			8.11	
			CCNE1			8.26	
6	NET G1	Mutant	ETV4	c.94C > A	p.L32M		2.3
			MTOR	c.77C > T	p.A26V		
7	NET G1	Mutant	AURKB	c.278 T > C	p.L93S		2.3
			ARID1A	c.6315_6316del	p.L2106Gfs*43		
8	NET G1	Mutant	DLL3	c.544_546del	p.A182Rfs*59		3.4
			FGFR3	c.1900G > A	p.A634T		
			DDR2	c.371G > A	p.R124Q		
9	NET G1	SV	KDM6A	IGR (downstream EXD1) ~ KDM6A:exon18			0.0
			EXD1				
10	NET G2	Germline	BRCA1	c.1171G > T	p.E391*		2.3
		Mutant	FLT1	c.2984C > G	p.T995S		
			XRCC1	c.521G > A	p.R174H		
11	NET G2	Mutant	CHD4	c.2686C > T	p.H896Y		4.6
			EPHA3	c.1545_1566del	p.G516Sfs*22		
			RB1	c.188_213del	p.K63Rfs*38		
			BAP1	c.1201_1212del	p.Y401_D404del		
12	NEC	Mutant	DOT1L	c.598C > T	p.R200C		10.3
			ERBB4	c.1538G > C	p.W513S		
			MAP2K4	c.1040 + 2 T > C			
			LRP1B	c.12797C > A	p.S4266*		
			TP53	c.1015G > T	p.E339*		
			MTOR	c.5126G > A	p.R1709H		
			INPP4B	c.2392G > T	p.E798*		
			ARID2	c.3029A > C	p.K1010T		
			RNF43	c.147_148del	p.R49Sfs*25		
		CNV	SMAD4			0.5	
13	NEC	Mutant	TP53	c.559 + 1G > C			5.7
			SMAD4	c.149A > G	p.K50R		
			AMER1	c.2096G > A	p.R699H		
			IGF1R	c.3532 T > A	p.W1178R		
			FLCN	c.444C > G	p.H148Q		
		CNV	MCL1			2.42	
			SDHB			0.5	
			RB1			0.45	
			BRCA2			0.56	

Abbreviations: AA, amino acid; CNV, copy number variation; SV, structural variation; TMB, tumor mutational burden.

### 3.6 Copy number variations, structural variations, and MSI of the 13 GI‐NENs

Structural variations (SVs) and Copy number variations (CNVs) were included for analysis. Two SVs had been found in two patients. These were *LZTR1‐BRD1* fusion in NEC patient and *KDM6A‐EXD1* fusion in NET G1 patient. Deep loss or high‐level amplification were found exclusively in NECs, and rare in NETs. Only *STK11* loss was found in NET patients. *SMAD4* (1/3, 33.3%), *SDHB* (1/3, 33.3%), *RB1* (1/3, 33.3%), and *BRCA2* (1/3, 33.3%) were found to be lost in NECs. *CRKL* (1/3, 33.3%), *CCNE1* (1/3, 33.3%), and *MCL1* (1/3, 33.3%) were found to be amplified in NECs. MSI was analyzed, but all tumors were microsatellite stable (MSS).

### 3.7 Altered signaling pathways involved in GI‐NENs cases

**FIGURE 5:**
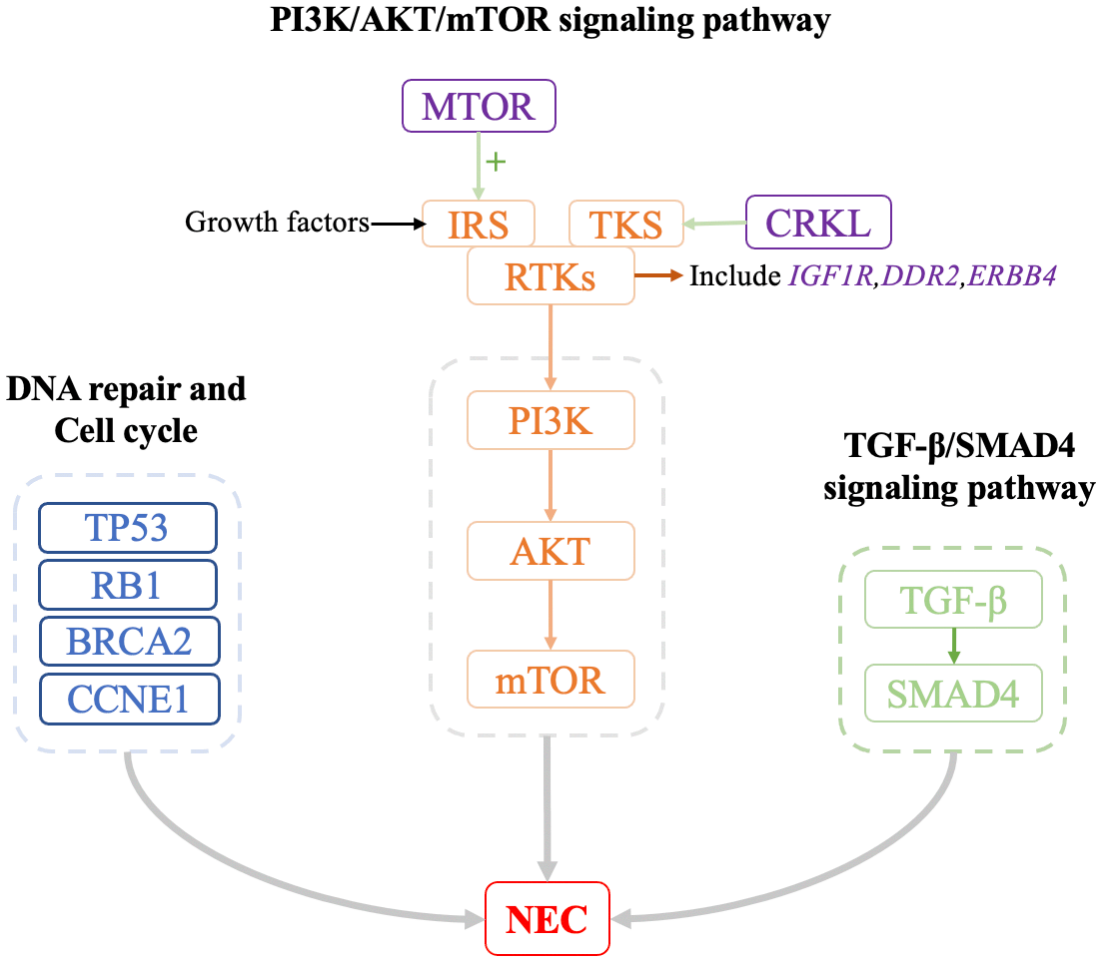
A simplified graph about GI‐NECs gene variations involved three signaling pathways. GI‐NECs gene variations involve regulation of DNA repair and cell cycle, PI3K/AKT/mTOR signaling pathway, and TGF‐β/SMAD4 signaling pathway. AKT, protein kinase B; IRS, insulin receptor substrate; mTOR, mammalian target of rapamycin; PI3K, phosphatidylinositol 3‐kinase; RTKs, protein tyrosine kinases; SMAD4, Recombinant mothers against decapentaplegic homolog 4; TGF‐β, transforming growth factor‐β; TKS, Tyrosine kinase substrate.

Gene variations involved in signaling pathways were integrated. Three pathways were found to be frequently altered in GI‐NENs. The first pathway was involved in regulation of DNA repair and cell cycle. The most common genetic alteration was *TP53* mutation, 100% (3/3) in NECs and 20% (2/10) in NETs. Mutations of *RB1*, *XRCC1*, and *BRCA1* were found in NETs (1/10, 10%, respectively), and loss of *RB1* as well as *BRCA2* in NECs (1/3, 33.3%, respectively). *CCNE1* copy number amplification was found in NECs (1/3, 33.3%). The second signaling pathway was Phosphatidylinositol 3‐kinase (PI3K)/protein kinase B (AKT)/mammalian target of rapamycin (mTOR). IGF1R, CRKL, DDR2, ERBB4, and mTOR are the important factors of this pathway. The third pathway was the TGF‐β/SMAD4 signaling pathway. Alterations of *SMAD4* have been found in NECs (2/3, 66.7%). The gene variations frequently involved in the respective signaling pathways of NECs are integrated in Figure 5.

## 4 DISCUSSION

GI‐NENs are rare cancers of the digestive tract in terms of prevalence. In recent years, some available therapeutic strategies for advanced gastroenteropancreatic NENs (GEP‐NENs) have been expanded considerably. Fundamental genomic, epigenomic, and transcriptomic differences were observed in GEP‐NETs with different primary sites and variable degrees of differentiation.^11^ Nonetheless, tumor grades and stages are decisive factors for the clinical treatment selection and prognosis estimation of NEN patients, despite the significant genetic heterogeneity of the tumor. Thus, a better understanding of underlying genetic characteristics is crucial, and novel biomarkers to enable individualized therapeutic strategies for clinical need are necessary. In this study, we aimed to provide additional data of the genetic characteristics of GI‐NENs and find the genomic alterations with potential clinical value.

Based on histopathology, mitotic count and Ki‐67 proliferation index, GI‐NENs are classified as NETs (G1, G2, and G3)and NECs in the latest WHO classification of tumors.^2^ Although NET G3 and NECs belong to high‐grade tumors, NET G3 tends to progress indolently, and the majority of NECs present with high clinical aggressiveness. In our cohort, two NET G3 cases presented with recurrence and metastasis, but the survival time was more than 5 years in both cases. In contrast, all NEC patients relapsed and metastasized quickly, and their average survival time was only 15.3 months.

The key IHC markers of GI‐NENs are PCK, CgA, Syn, INSM‐1, and CD56. In this study, the positive rate was 100% (PCK), 92.3% (Syn), 84.6% (CD56), 84.6% (INSM1), and 53.4% (CgA). No case was positive for ATRX and DAXX.

Previous studies have reported that the genetic characteristics of NECs in colorectum were more similar to that of colorectal adenocarcinoma, and the mutations of *TP53*, *APC*, *KRAS*, *BRAF*, and *RB1* are the most common,^4^, ^5^ and *TP53* mutations were not observed in well differentiated pancreatic NETs.^12^, ^13^ Yachida et al. proved that *TP53* alterations are common in NECs of gastrointestinal system.^14^ In our study, the *TP53* mutation rate was 100% (3/3) in NEC patients, 33.3% (2/6) in patients with NET G2 and G3, 0% (0/4) in NET G1 patients. These results were consistent with Yachida et al.^14^ *TP53* mutation rate of NEC was significantly higher compared to NET, and gradually increasing with the degree of differentiation of GI‐NENs. P53 immunoreactivity tested by IHC, an alternative method of *TP53* mutation testing, was observed as an overexpression in pancreatic NECs, and negative or lower expression was detected in pancreatic NETs. Combined with expression of RB1, DAXX, and ATRX, P53 may be used as indicator for distinction between NET and NEC.^15^ Still, in our study, P53 was negatively tested by IHC in two NEC patients with *TP53* mutation. P53 expression was completely absent in patients with the *TP53* mutation, consistent with the “null” pattern of aberrant/mutation‐type P53 expression. In our study, we found two NEC patients with the *TP53* mutation of stop‐gained mutation or frameshift mutation, which reslutes in P53 is not tested by the P53 antibody. These results were found in small cell NEC of the uterine cervix also,^16^ so P53 immunoreactivity was not always consistent with TP53 alterations. Negative P53 expression tested by IHC as the indicator of differential diagnosis between NETs and NECs of GI is unreliable. *RB1* mutation was found in NETs (1/10, 10%) and loss of *RB1* CNV was found in NECs (1/3, 33.3%). Yachida et al. reported *RB1* alterations are common in GI‐NECs as well.^14^ Therefore, *RB1* mutation may also be a molecular event of NENs development. In addition, most commonly reported genetic variations of *APC*, *KRAS*, and *BRAF* of colorectal NECs in a previous study, were not found in any NEC‐patient in our study.

Currently, immunotherapies, inhibitors of various signaling pathways, and apoptosis promoters have been approved by US Food and Drug Administration (FDA), or standard care biomarkers recommended by the National Comprehensive Cancer Network (NCCN). Accordingly, these potential targeted therapies were applicated in GI‐NEN patients dependent on identification of genetic changes that are vulnerable to such therapies.^16^ In our study, we found *BRCA1/2* and *ATM* gene alterations of GI‐NEN. Alterations of *BRCA1/2* and *ATM* may lead to homologous recombination deficiency (HRD), increasing the risk of breast‐, ovarian‐, prostate‐, and other cancers.^17^, ^18^ Poly (ADP‐ribose) polymerase (PARP) inhibitors of BRCA1/2 and ATM, such as Olaparib, have been approved by US FDA for breast cancer and ovarian cancer treatment. Recently, Stopsack KH, et al. showed that prostate cancers harboring BRCA and ATM alterations may potentially benefit from Olaparib.^19^ Thus, we suspect that PARP inhibitors may serve as a potential treatment strategy for advanced GI‐NENs with *BRCA1/2* and *ATM* mutation.

In our study, we found a *CRK*‐like (CRKL) amplification in NEC patients (1/3, 33.3%). Previous studies demonstrated that overexpression of CRKL enhances tumor cell functions, such as proliferation, invasion, migration, epithelial mesenchymal transformation, cell growth, and metastasis, which correlated with poor prognosis in many types of human cancers.^20^, ^21^, ^22^ CRKL overexpression may be a cancer‐specific event and serves as a good therapeutic target.^19^ *CRKL* amplification may enhance CRKL expression, play an important role in GI‐NEC development and act as the targetable biomarker for treatment. This is the first report of *CRKL* alteration in NEC. Of course, more research is needed to confirm this speculation.

We found *IGF1R*, *DDR2*, *ERBB4*, and *MTOR* alterations in NEC patients. They are the key factors in the PI3K/AKT/mTOR pathway. Banck et al. found mutually exclusive amplification of AKT1 or AKT2 in PI3K/Akt/mTOR signaling was the most common event in patients with small intestinal NETs.^23^ We could not find these alterations, speculating the molecular events are different in NENs of various sites. The PI3K/AKT/mTOR pathway is an important contributor to basic biological functions of cell growth, apoptosis, transformation, and metabolism.^24^ The PI3K/AKT/mTOR is suggested to act as an essential part in the tumor's development and its potential as new therapeutic target. Inhibitors of PI3K/AKT/mTOR were studied and some were used in clinical trials.^24^, ^25^, ^26^, ^27^ We believe that NEC patients with these gene alterations may possibly benefit from the inhibitor treatment of PI3K/AKT/mTOR.

*SMAD4* alteration was found in NECs (2/3, 66.7%) in our study. *SMAD4* is one of the major driver genes for pancreatic cancer, its mutation makes pancreatic cancer resistant to radiotherapy.^28^ *SMAD4* is a key transcriptional factor of TGF‐beta pathway and acts as a tumor suppressor gene in colorectal cancer.^29^ Thus, we suggest that the alteration of *SMAD4* is a contributor to NEC development. *CCNE1* amplification is correlated to metastasis in gastric carcinoma and gynecologic high‐grade serous carcinoma, *poor prognosis in lung adenocarcinoma*. Moreover, it may be a predictive biomarker of chemotherapy resistance in epithelial ovarian cancer.^30^, ^31^, ^32^, ^33^ Previous studies reported that *CCNE1* gene amplification of gastric MiNENs occurs in up to 18%–50%, resulting in an increase of protein expression,^34^, ^35^ and NECs with intact Rb demonstrated mutually exclusive amplification of CCNE1 or MYC.^12^ Here, we found *CCNE1* gene amplification (1/3, 33.3%) in gastric NEC patients with no RB alteration and MYC amplification. MCL1 is an anti‐apoptotic BCL2 family member that is often overexpressed in various malignant tumors.^36^ In the present study, *MCL1* amplification (1/3, 33.3%) was found in NEC patients. We suspect that gene amplification of *CCNE1* and *MCL1* may promote progress and relate to poor prognosis of GI‐NEC. CCNE1 and MCL1 may be possible markers for treatment choice and prognosis assessment. Of course, these genes have rarely been reported previously, and these results need more research to confirm, because only a small number of cases were tested for these genes.

In our study, we found TMB of NEC patients is more than five. There were significant differences regarding TMB between the NET and NEC patients (*p* = 0.0009). These results were consistent with Riet et al.^37^ They observed relatively high TMB in NEC (average 5.45 somatic mutations per megabase) with TP53.^37^ All NEC patients relapsed and metastasized in a short time, and their average survival time was only 15.3 months. Considering the poor prognosis of advanced NECs and the limited therapy options, it is worth trying immunotherapy, because high TMB was demonstrated to be related to better efficiency of anti‐programmed cell death 1 (PD‐1)/programmed death ligand 1 (PD‐L1) therapy.^38^, ^39^ A previous study indicated a higher MSI‐H status in high‐grade NENs, compared to low‐grade tumors, regardless of the tumor location.^7^ Colorectal NEC with MSI‐H status had similar frequency as MSI‐colorectal adenocarcinoma and resembled their molecular profile and pathogenesis.^40^ However, in our study we did not find MSI in all patients.

In this study, NGS analysis identified genetic alterations in GI‐NENs, such as *BRAC1/2*, *ATM*, *MCL1*, *CRKL*, *CCNE1*, *IGF1R*, *DDR2*, *ERBB4*, and *MTOR* mutations. There were significantly different numbers of gene alterations between NETs and NECs. We found extensive mutations and CNVs in NECs. The TMB of NECs is significantly higher than that of NETs. These genetic alterations involved PI3K/AKT/mTOR, TGF‐β/SMAD4, and DNA repair and cell cycle pathways. Previous studies had demonstrated that these genes are oncogenes or tumor suppressor genes, and inhibitors of some of these genes have been approved to be applicable in clinics. The present study has provided the unprecedented opportunity to investigate genomic and molecular alterations in different histological types of GI‐NENs. Consequently, the presence of genetic variations that are vulnerable to potential individualized therapies are definitely relevant, especially for patients with high grade GI‐NENs. Melone et al. showed ATM‐dependent signaling was the most significant pathways in NEN patients of thyroid, pancreas, intestine, and lung by multi‐omics analysis.^41^ In our study, *BRAC1/2* and *ATM* alterations of ATM‐dependent signaling were found in GI‐NENs also.

Due to some limitations of this study, there is still need for improvement. First, relatively few cases were included, thus, the results for GI‐NEN were difficult to generate. Second, genetic detection was confirmed by a commercial panel with limited genes selection. Lastly, the functions of genetic alterations for GI‐NEN development and personal therapies need further validation and investigation.

## AUTHOR CONTRIBUTIONS

**Qiong Dai:** Data curation (equal); formal analysis (equal); investigation (equal); resources (equal); writing – original draft (equal). **Jinping Zhang:** Data curation (equal); formal analysis (equal); investigation (equal); resources (equal); writing – original draft (equal). **Weili Long:** Data curation (equal); investigation (equal); resources (equal). **Johannes Haybaeck:** Supervision (equal); validation (equal); writing – review and editing (supporting). **Zhihui Yang:** Funding acquisition (lead); supervision (equal); validation (equal); writing – original draft (equal); writing – review and editing (equal).

## FUNDING INFORMATION

This study was supported by the Project of Department of Science and Technology, Sichuan province (2018JY0398), and the Sichuan Science and Technology Support Project (22ZDYF3780).

## CONFLICT OF INTEREST

The authors have no competing interests.

## ETHICS APPROVAL AND CONSENT TO PARTICIPATE

The authors state that the research has obtained approval by the ethics committee of the Affiliated Hospital of Southwest Medical University of China. All researches were performed in accordance with relevant guidelines. The informed consent has been obtained from all participants.

## CONSENT FOR PUBLICATION

All authors state that they consent to publish the paper in cancer medicine.

## Data Availability

I confirm that my article contains a Data Availability Statement even if no data is available (list of sample statements) unless my article type does not require one.
